# Anthropometric Parameters in Patients with Fatty Acid Oxidation Disorders: A Case–Control Study, Systematic Review and Meta-Analysis

**DOI:** 10.3390/healthcare10122405

**Published:** 2022-11-30

**Authors:** Maria Wasiewicz-Gajdzis, Małgorzata Jamka, Jakub Geltz, Kamila Bokayeva, Łukasz Kałużny, Joanna Jagłowska, Jarosław Walkowiak

**Affiliations:** 1 Department of Pediatric Gastroenterology and Metabolic Diseases, Poznan University of Medical Sciences, Szpitalna 27/33, 60-572 Poznan, Poland; 2 Department of Pediatrics, Hematology and Oncology, Medical University of Gdansk, Dębinki Str. 7, 80-211 Gdansk, Poland

**Keywords:** fatty acid oxidation disorder, FAOD, BMI

## Abstract

This study compared the anthropometric parameters of patients with fatty acid oxidation disorders (FAOD) and healthy controls, showing an increased prevalence of abnormal body weight (overweight and obesity) in the FAOD group. First, differences in BMI, BMI percentiles and z-scores, and weight and weight percentiles were compared in a cohort of 39 patients with FAOD and 156 healthy controls, as well as between patients born before and after the introduction of a populational newborn screening programme (NBS) in 2014 in Poland. We also performed a systematic literature review yielding 12 studies mentioning anthropometric parameters in 80 FAOD patients and 121 control subjects, followed by a meta-analysis of data from 8 studies and our cohort. There were significant differences in body weight percentiles (*p* = 0.001), BMI (*p* = 0.022), BMI percentiles (*p* = 0.003) and BMI z-scores (*p* = 0.001) between FAOD patients and controls in our cohort but not between pre- and post-newborn-screening patients. The meta-analysis did not show any differences in weight and BMI in all tested subgroups, i.e., all FAOD patients vs. controls, medium-chain acyl-CoA dehydrogenase (MCADD) patients vs. controls and patients with FAOD types other than MCAD vs. controls. These results, however, should be interpreted with caution due to the overall low quality of evidence as assessed by GRADE, the small sample sizes and the significant heterogeneity of the included data.

## 1. Introduction

Fatty acid oxidation disorders (FAOD) are rare autosomal recessive inborn errors of metabolism resulting in defective mitochondrial beta-oxidation or carnitine-mediated transport of fatty acids into the mitochondria. The prevalence of FAOD ranges from 1:20,000 for medium-chain acyl-CoA dehydrogenase deficiency (MCADD) [1] to 1:2,000,000 for multiple acyl CoA dehydrogenase deficiency (MADD). FAOD present with a broad spectrum of symptoms, such as hypotonia, recurrent rhabdomyolysis, exercise intolerance, hypoketotic hypoglycaemia, liver failure and sudden infant death syndrome [2]. Presentation depends on the type of defect, with MCADD tending to have a milder phenotype. At the same time, long-chain fatty acid oxidation disorders (LC-FAOD) and carnitine metabolism disorders present more severe symptoms. Complications include retinopathy, acute or chronic cardiomyopathy and polyneuropathy [3]. The treatment recommendations are based on experts’ consensus and include avoidance of fasting and strenuous exercise, aggressive treatment of infections, and in some cases, carnitine supplementation [2]. Most patients, especially with LC-FAOD, require dietary modifications such as diets with low LCT content, MCT oil supplementation and night feeds [4]. In the past, a high-carbohydrate diet was also recommended. Some of the research published in previous decades suggests a tendency towards overweight and obesity [5,6] among FAOD patients; however, the lack of control groups hinders conclusion.

We hypothesised that patients with FAOD, especially LC-FAOD, are at increased risk of excess weight and obesity due to the treatment modalities and lifestyle modifications mentioned above. Therefore, various anthropometric data were analysed to investigate whether FAOD patients differ from the general population regarding weight and BMI. The analysis included a cohort of FAOD patients from two metabolic centres in Poland and patients born before and after the introduction of a populational newborn screening programme (NBS) in 2014 in Poland [7], as well as a systematic review and meta-analysis of studies related to anthropometrics in patients with FAOD.

## 2. Materials and Methods

### 2.1. Case–Control Study

The study protocol was approved by the Poznań University of Medical Sciences Ethical Committee, and all procedures were performed according to the Helsinki Declaration of 1975 [8]. The study was conducted per the STROBE guidelines [9].

#### 2.1.1. Study Population

The medical records of 39 FAOD patients from two metabolic centres in Poland (22 patients from the Department of Paediatric Gastroenterology and Metabolic Diseases at Poznan University of Medical Sciences and 17 patients from the Department of Paediatrics, Haematology and Oncology, Medical University of Gdansk) were reviewed retrospectively to retrieve age, sex and anthropometric data (weight, height, BMI). FAOD diagnosis was confirmed in all patients with blood acylcarnitine assays, urinary organic acid profiles and genetic testing. Patients in unstable clinical conditions were excluded from the study. Sex- and age- matched control subjects were recruited using the propensity score matching method. The inclusion criteria included age 0–18 years, good general health, lack of chronic disease (such as asthma, diabetes, hypertension, hypothyroidism) and documented intellectual or physical disability. Children born prematurely (before 37 weeks of gestation) were also excluded. Controls data were obtained in July 2022 by a review of the charts from a GP’s office located in a socially diverse neighbourhood in Poznań, Poland. The included data were recorded during routine health check-ups and immunisation visits. Percentiles and z-scores were calculated using the PediTools Software [10], which is based on WHO Child Growth Standards for children aged 0–2 [11] and CDC Clinical Growth Charts for children and adolescents aged 2–20 years [12].

#### 2.1.2. Anthropometric Measurements

Measurements were recorded between May 2018 and July 2022 by treating physicians during routine clinical check-ups, in the morning, in light clothing and without shoes, using Seca 703 scales equipped with a measuring rod (Hamburg, Germany) for children > 2 years of age, and Seca 376 scales equipped with a measuring rod (Hamburg, Germany) for children < 2 years of age. The most recent set of measurements for each patient was used in the study. The weight measurements had an accuracy of 0.1 kg, while height was measured with an accuracy of 0.1 cm. BMI was calculated using the obtained values, with an accuracy of 0.1 kg/m^2^.

#### 2.1.3. Statistical Analysis

Continuous data are presented as means (M) with standard deviations (SD), medians (Me) and interquartile ranges. Categorical variables are presented as absolute and relative frequencies. Normality was assessed with the Shapiro–Wilk test. Due to the non-normal distribution of the data for most studied variables, the anthropometric parameters of patients and controls and pre- and post-NBS patients were compared using the nonparametric U-Mann–Whitney test. ANOVA Kruskal–Wallis analysis and posthoc tests were used to compare different types of FAOD with the control group, and the Chi-squared statistic was used for categorical data. A *p*-value < 0.05 was considered statistically significant. Statistica Software version 13 (TIBCO Software Inc., Palo Alto, CA, USA) was used for the statistical calculations.

### 2.2. Systemic Review and Meta-Analysis

#### 2.2.1. Protocol and Registration

The systematic review and meta-analysis were carried out and reported following the Preferred Reporting Items for Systematic Reviews and Meta-Analyses (PRISMA). Prisma Checklist is presented in Supplementary Materials (Table S3). The study protocol was registered in the international prospective register of systematic reviews (PROSPERO) database with ID number CRD42022343364.

#### 2.2.2. Search Strategy

The following databases were searched: PubMed (Medline), Embase, Web of Science and Scopus; we used the following search terms: fatty acid oxidation disorders FAOD; long-chain 3-hydroxyacyl-coenzyme A dehydrogenase deficiency, LCHADD; medium-chain acyl-CoA dehydrogenase deficiency, MCADD; very long chain acyl-CoA dehydrogenase deficiency, VLCADD; short-chain acyl-CoA dehydrogenase deficiency, SCADD; carnitine palmitoyltransferase II deficiency, CPTIID or CPT 2D; mitochondrial trifunctional protein deficiency, TFPD. Searches were restricted to titles and abstracts, the English language and studies performed in humans. No date limits were applied. In addition, the articles were reviewed for anthropometric data (weight, BMI and percentiles and z-scores for these parameters), and the reference lists of the identified papers were checked for articles that database searches might have missed.

The search strategy was as follows:

PubMed (Medline)

#1 (disease name, i.e., LCHADD [Title/Abstract])

#2 AND (English and Human [Filter])

Embase

#1 (disease name i.e., LCHADD [Candidate Term])

#2 AND (Abstract and English and Human [Filter])

Web of Science

#1 (disease name, i.e., LCHADD [Topic])

#2 AND (English [Filter])

Scopus

#1 (disease name, i.e., LCHADD [Article title, Abstract, Keywords])

#2 AND (English and Human [Filter])

#### 2.2.3. Eligibility Criteria

The following inclusion criteria were applied:

1. Study type: observational (case–control, cohort, case series) or experimental studies (any type); a study was excluded when only an abstract was available.
2. Language: English.
3. Study population: children (>1 month of age) and adults with confirmed diagnosis (biochemically or genetically) of fatty acid oxidation disorder (LCHADD, MCADD, VLCADD, SCADD, CPT IID or TFPD).
4. Outcomes: anthropometric parameters (weight, BMI and percentiles and z-scores for these parameters).

Exclusion criteria:

1. Study type: letters, case studies, conference abstracts, non-human studies.
2. Language: papers published in a language other than English.
3. Population: newborns (age < 1 month), pregnant and breastfeeding women, patients in unstable or critical clinical condition.

#### 2.2.4. Study Selection Process

Three researchers (MWG, JG and KB) searched each database independently. First, the reviewers checked titles, abstracts and then full texts based on the eligibility criteria. Studies deemed appropriate by at least one of the reviewers were incorporated in the next step, with any disagreements resolved by consensus within the review team.

#### 2.2.5. Data Extraction

Two reviewers (MWG and JG) independently performed data extraction, and two others (MJ and JW) checked it. The corresponding authors were contacted for missing information. The following information was extracted from each paper:

1. General information: full title of the article, list of authors, country, journal name, year of publication.
2. Study characteristics: study design (experimental or observational).
3. Population characteristics: number of participants in patient groups and control groups (if applicable), types of FAOD diagnosed in patients, age of the participants and sex of the participants.
4. Outcomes recorded: anthropometric parameters of patients and controls (if present)—weight, BMI and percentiles and z-scores for these parameters.

#### 2.2.6. Certainty of Evidence Assessment

Grading of Recommendations, Assessment, Development, and Evaluation (GRADE) was used to estimate the quality of the included data [13]. Two independent researchers (MWG and MJ) conducted the assessment, and any disagreements were resolved by discussion.

#### 2.2.7. Statistical Analysis

The meta-analysis was performed using Comprehensive Meta-Analysis version 3.0 software (Biostat, Inc. Englewood, CO, USA). A *p*-value < 0.05 was considered as statistically significant. In some studies, M and SD were calculated based on individual patients’ and control subjects’ data. The most recent data set was used if several data points were available for the patient. For the meta-analysis, two scenarios for patient groups were considered. In the first one, all patients with diagnosed FAOD were put in one group and analysed vs. controls. In the second one, the patients were divided into an MCAD group and an other-types-of-FAOD group and analysed vs. controls. The meta-analysis was performed if a given outcome was reported in at least two studies. There was no need to unify the data, as they were presented in SI units in all included studies. The analyses were undertaken to compare weight and BMI between patients with FAOD vs. control subjects. Data analysis was performed, including calculating the effect of sizes with 95% CI, using either random-effect models (to analyse studies with moderate to high heterogeneity) or fixed-effect models (to analyze outcomes without heterogeneity). Mean differences (MD) were used as a summary statistic to facilitate comparing outcomes between groups. Forest plots were generated to visualise the study-specific effect sizes, with 95% CI. Funnel plots were generated, and Begg’s and Egger’s tests were performed to assess publication bias. Heterogeneity between studies was evaluated using Cochran’s Q test, with *p* < 0.1 indicating significant heterogeneity. The I^2^ test was used to assess consistency between studies, with an I^2^ value of <30% indicating a low risk of heterogeneity, 30–75% indicating a moderate risk of heterogeneity, and >75% indicating a high risk of heterogeneity. Sensitivity analysis and cumulative analysis were also performed.

## 3. Results

### 3.1. Case–Control Study Results

The population characteristics are summarised in Table 1, showing no differences in age and sex structure between the groups. The FAOD cohort included 19 patients with MCADD (48.7%), 14 patients with LCHADD (35.9%), 5 patients with VLCADD (12.8%) and one patient with CDSP (2.6%). In total, 17 patients (43.6%) were born before the introduction of NBS in Poland, while 22 patients (56.4%) were born after it. We observed statistically significant differences in body weight percentiles (*p* = 0.001), BMI (*p* = 0.022), BMI percentiles (*p* = 0.003) and BMI z-scores (*p* = 0.001), with FAOD patients having larger body weight and BMI than healthy controls. There were no statistically significant differences in weight percentiles (*p* = 0.702), BMI percentiles (*p* = 0.590) and BMI z-scores (*p* = 0.365) observed for pre- and post-NBS patients. The Kruskal–Wallis test was conducted to determine differences in anthropometric parameters between different types of FAOD patients and controls, showing statistically significant differences in weight centiles (*p* = 0.009), BMI centiles (*p* = 0.044) and BMI z-scores (*p* = 0.021). There were no differences in weight (*p* = 0.164) and BMI (*p* = 0.260), but the posthoc analysis confirmed differences in weight centiles between MCAD and the control group (*p* = 0.036).

### 3.2. Results from the Systematic Review and Meta-Analysis

#### 3.2.1. Search Results

**Figure 1:**
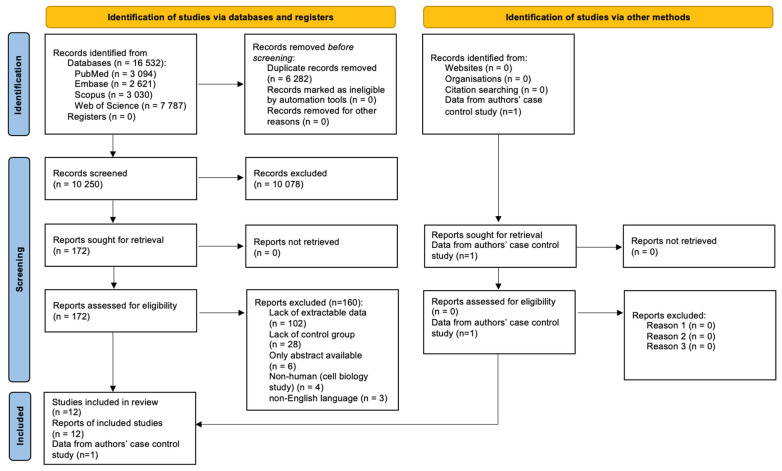
PRISMA 2020 Flow Diagram.

Figure 1 shows the results of the search identifying a total of 16,532 records, of which, 6282 were duplicates. The titles and abstracts of the following 10,250 records were screened, and 172 full texts were retrieved, of which, 12 articles were included in the study.

However, the studies by McCoin et al., 2019, McCoin et al., 2016 and Gillingham et al., 2013 [14,15,16] were conducted in the same centre on overlapping populations; therefore, we decided to include a study by McCoin et al., 2016 in the meta-analysis, since it had the largest study and control populations. Of the remaining papers, seven had extractable data on the study and control groups [17,18,19,20,21,22]; therefore, eight papers were incorporated into the meta-analysis along with our case–control study.

#### 3.2.2. Reported Anthropometric Parameters

Weight was reported in seven studies [14,16,18,19,21,22,23]. The data on weight were presented in the form of individual patient data, M, SD, Me and Z-score Me and Z-score SD. BMI was reported in 10 papers [14,15,16,17,18,19,20,23,24,25]. The data were presented as individual patient data, M, SD, Me, Z-score Me and Z-score SD.

#### 3.2.3. Characteristics of the Included Studies

The characteristics of the included studies are presented in Table 2, and the data from the included studies are presented in Table 3. Characteristics and data from studies without control groups that contained data on anthropometric parameters, but were not included into the meta-analysis, are presented in Tables S1 and S2 in Supplementary Materials.

All the included 12 papers were observational studies. Seven studies were performed in Europe, four in the Netherlands [18,19,22,24], two in Denmark [17,25], one in Spain [23], three in North America (USA) [14,15,16], one in Asia (Japan) [20] and one in Australia [21].

#### 3.2.4. Characteristics of the Study Participants

A total of 201 participants were included in the studies, of which, 80 were FAOD patients, while 121 were control subjects. The study size varied from 6 to 24 participants. In nine studies, children were incorporated into the study groups. Among the included FAOD patients, 16 had MCADD, 14 had VLCADD, 33 had LCHADD, 9 had CPT2D, 1 had CPT1D, 3 had MADD, and four had SCADD. FAOD diagnosis was confirmed via biochemical or genetic testing. In one study, the patients were (mostly) diagnosed by the NBS [23], one study stated that the patients were diagnosed before the NBS programme [24], and ten papers included no information on the NBS status. As mentioned above, some of the study groups were overlapping.

### 3.3. Comparison of Weight between FAOD Patients and Controls

**Figure 2:**
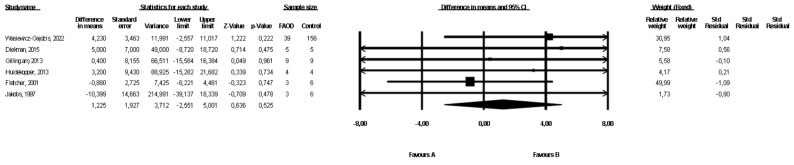
Forest plot—comparison of body weight in FAOD patients vs. controls [16,18,19,21,22].

Six studies presented data on weight for the FAOD and the control groups ([16,18,19,21,22], Wasiewicz-Gajdzis et al., 2022). No study reported statistically significant differences between the groups. The meta-analysis showed no difference in weight between the FAOD and the control groups (fixed-effects model, SE: 1.93, 95% CI: −2.55, 5.00, *p* = 0.53, Figure 2). The risk of heterogeneity was assessed as small (Q value = 0.232, *p* = 0.80, I^2^ = 0). The funnel plot of standard error by a difference in means of weight is presented in the Supplementary Materials (Figure S1). The Begg and Mazumdar’s rank correlation test (*p* = 0.70) and the Egger’s regression test (*p* = 0.96) were performed.

### 3.4. Comparison of BMI between FAOD Patients and Controls

**Figure 3:**
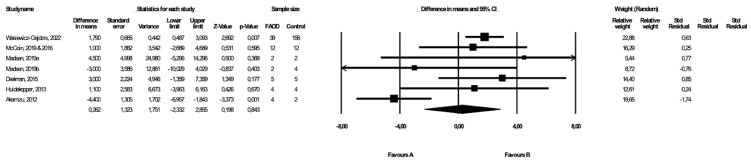
Forest plot—comparison of BMI in FAOD patients vs. controls [14,15,17,18,19,20,25].

BMI was compared between FAOD patients and control subjects in seven studies ([14,17,18,19,20,25], Wasiewicz-Gajdzis et al., 2022), with a statistically significant difference in BMI in two studies ([20], Wasiewicz-Gajdzis et al., 2022). The meta-analysis showed no difference in BMI between FAOD and control groups (random-effects model, SE: 1.32, 95% CI: −2.33, 2.86, *p* = 0.843, Figure 3), but there was a substantial risk for heterogeneity (Q value = 20.73, *p* = 0.00, I^2^ = 71.05). The funnel plot of standard error by a difference in means of BMI is presented in the Supplementary Materials (Figure S2); the Begg and Mazumdar’s rank correlation test (*p* = 0.76) and the Egger’s regression test (*p* = 0.73) were also performed.

### 3.5. Comparison of Weight between MCAD Patients and Controls

**Figure 4:**
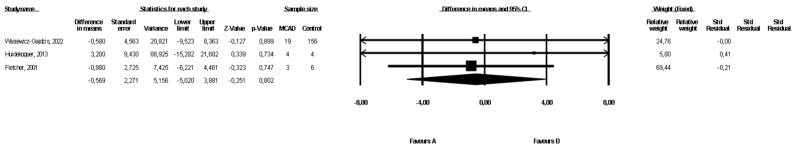
Forest plot—comparison of bodyweight in MCAD patients vs. controls [19,20].

The weight of the MCAD patients and controls was compared in three studies ([19,21], Wasiewicz-Gajdzis et al., 2022) with no study reporting a statistically significant difference in weight. The meta-analysis showed no difference in weight between MCAD patients and the control group (fixed-effects model, SE: 2.27, 95% CI: −5.02, 3.88, *p* = 0.80, Figure 4), and there was a small risk of heterogeneity (Q value = 0.17, *p* = 0.91, I^2^ = 0). The funnel plot of standard error by a difference in means of BMI is presented in the Supplementary Materials (Figure S3); the Begg and Mazumdar’s rank correlation test (*p* = 0.30) and the Egger’s regression test (*p* = 0.23) were also performed.

### 3.6. Comparison of BMI between MCAD Patients and Controls

**Figure 5:**
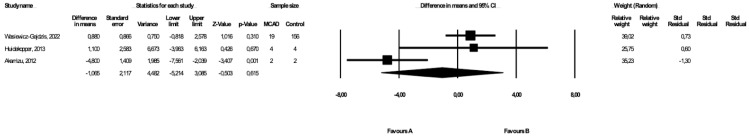
Forest plot—comparison of BMI in MCAD patients vs. controls [19,20].

Three studies presented data on BMI in MCAD patients and controls ([19,20], Wasiewicz-Gajdzis et al., 2022), with no study reporting a statistically significant difference in BMI. The meta-analysis showed no difference in BMI between the MCAD patients and the control group (random-effects model, SE: 2.12, 95% CI: −5.21, 3.09, *p* = 0.62, Figure 5). The risk of heterogeneity was considered small (Q value = 12.23, *p* = 0.002, I^2^ = 83.65). A funnel plot of standard error by a difference in means of BMI is presented in the Supplementary Materials (Figure S4). The Begg and Mazumdar’s rank correlation test (*p* = 1.00) and the Egger’s regression test (*p* = 0.76) were also performed.

### 3.7. Comparison of Weight between Patients with Types of FAOD Other Than MCAD and Controls

**Figure 6:**
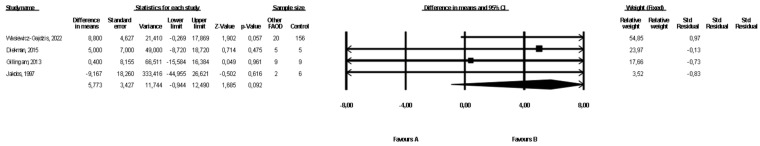
Forest plot—comparison of body weight in patients with FAOD other than MCAD vs. controls [16,18,22].

Data on the weight of patients with FAOD types other than MCAD were available in four studies ([16,18,22], Wasiewicz-Gajdzis et al., 2022), with no study reporting a statistically significant difference in weight. The meta-analysis showed no difference in weight between patients and the control group (fixed-effects model, SE: 3.43, 95% CI: −0.94, 12.49, *p* = 0.09, Figure 6), and the risk of heterogeneity was assessed as small (Q value = 1.54, *p* = 0.68, I^2^ = 0). A funnel plot of the standard error by a difference in means of weight is presented in the Supplementary Materials (Figure S5). We performed the Begg and Mazumdar’s rank correlation test (*p* = 0.09) and the Egger’s regression test (*p* = 0.05).

### 3.8. Comparison of BMI between Patients with Types of FAOD Other Than MCAD and Controls

**Figure 7:**
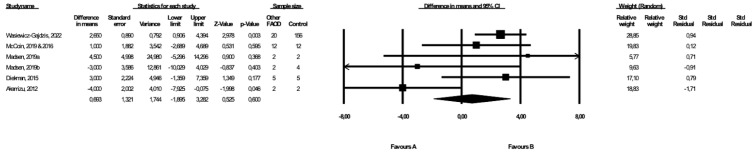
Forest plot—comparison of BMI in patients with FAOD other than MCAD vs. controls [14,15,17,18,20,25].

Six studies included data on the BMI of patients with types of FAOD other than MCAD ([14,17,18,20,25,26], Wasiewicz-Gajdzis et al., 2022), with no study reporting a statistically significant difference in BMI. The meta-analysis showed no difference in BMI between patients and the control group (random-effects model, SE: 1.32, 95% CI: −1.90, 3.26, *p* = 0.60, Figure 7), and the risk of heterogeneity was substantial (Q value = 11.67, *p* = 0.04, I^2^ = 57.17). A funnel plot of the standard error by a difference in means of weight is presented in the Supplementary Materials (Figure S6). We performed the Begg and Mazumdar’s rank correlation test (*p* = 0.71) and the Egger’s regression test (*p* = 0.42).

### 3.9. Sensitivity and Cumulative Meta-Analyses

The sensitivity and cumulative meta-analyses revealed no statistically significant differences, and the relevant figures are provided in the Supplementary Materials (Figures S6–S18).

### 3.10. Certainty of Evidence Assessment

The certainty in the evidence for each outcome is presented in Table 4.

## 4. Discussion

A case–control study and a systematic review and meta-analysis were conducted to compare anthropometric parameters between FAOD patients and healthy people. The case–control study revealed a statistically significant difference in weight percentiles, BMI, BMI percentiles and BMI z-scores between our FAOD cohort and healthy control subjects, with FAOD patients being heavier and having a higher BMI. However, there were no significant differences in anthropometric parameters of patients born pre- and post-NBS introduction in Poland. There were no differences in anthropometric parameters between patients with different FAOD diagnoses, either.

Our systematic review and meta-analysis revealed no differences in the anthropometric parameters of patients with all types of FAOD and controls. There was also no statistically significant difference in weight and BMI when comparing a group of people with MCAD and a group of patients with types of FAOD other than MCAD to healthy controls.

Some of the studies which were not included in the meta-analysis due to lack of extractable data yielded similar conclusions as our meta-analysis: Rücklová et al. observed good growth and weight control among patients [27], Rovelli et al. and Anderson et al. found that the anthropometric parameters of FAOD patients were not different from those of the general population [28,29]. Some of the older works described a failure to thrive [30,31] and an increased incidence of overweight and obesity [5,6,32]. Two papers reported increased adiposity in patients with FAOD [16,33].

Differences in treatment guidelines implementation, such as the extent of dietary restrictions, the maximal recommended duration of fasting, the implementation of night feeds, or enteral nutrition in metabolic centres, might have caused the differences in results in our case–control study and meta-analysis. There were also differences in healthcare services provided to patients that might affect the nutritional status; some studies described a close supervision of patients by dieticians [28], while such services were not widely available to patients in other centres. Furthermore, a fear of metabolic decompensation and complications could be differentiated depending on the care possibilities available to patients and personal experience, which might drive overfeeding and excessive avoidance of physical activity.

This is the first meta-analysis comparing the anthropometric parameters of patients with FAOD to those of healthy controls; the cohort from Polish centres was significant compared to other studies. The strength of this work is that it was based on a comprehensive search of four large databases: PubMed, Embase, Scopus and Web of Science, using broad search terms. Furthermore, the double-counting of patients from publications prepared in the same centres or by the same authors was prevented.

The major limitation of this study is the overall low level of evidence, as assessed by GRADE. Other limitations of our study comprise the small sample sizes in the included groups, which were due to the low prevalence of inborn errors of metabolism in the population. Furthermore, many studies incorporated children and adults, hindering the analysis. Another limitation was the lack of extractable data in publications for the systemic review, and the authors did not provide data when contacted. In addition, some publications had overlapping patient groups, and only a few publications considered wth control groups, which limited the number of papers that could be included in the meta-analysis. Due to a lack of research, we could not perform a meta-analysis to compare the effect of NBS on the anthropometric parameters. Therefore, the results from this study should be interpreted with caution. More research is needed to clearly establish the impact of FAOD on the nutritional state of patients.

## 5. Conclusions

There were statistically significant differences in anthropometric parameters between FAOD patients and healthy controls in our case–control study, with patients having higher body weight and BMI, but no differences were observed for pre- and post-NBS patients and patients with different types of FAOD. However, the meta-analysis showed no differences in anthropometric parameters between patients and controls, and the results must be interpreted cautiously due to the small sample sizes and heterogeneous data.

## Figures and Tables

**Table 1:** Population characteristics.

	FAOD n = 39	Controls n = 156	*p*
Age (years)			
mean ± SD	7.1 ± 4.4	7.1 ± 4.4	*p* = 1.000
median	5.8	5.6	
interquartile range	(3.3–10.5)	(3.2–10.2)	
Sex (n (%))			
Females	16 (41.0%)	64 (41.0%)	*p* = 1.000
Males	23 (59.0%)	92 (59.0%)	
Weight (kg)			
mean ± SD	32.1 ± 21.1	27.9 ± 18.9	*p* = 0.145
median	23.8	19.3	
interquartile range	(16.3–41.8)	(15.0–34.0)	
Weight percentile			
median	83	54	*p* = 0.001
interquartile range	(67–96)	(32–74)	
BMI (kg/m^2^)			
mean ± SD	18.3 ± 4.2	16.5 ± 3.6	*p* = 0.022
median	17.9	15.9	
interquartile range	(15.3–20.5)	(14.6–17.9)	
BMI percentile			
median	76	48	*p* = 0.003
interquartile range	(40–95)	(16–74)	
BMI z-score			
median	0.71	−0.07	*p* = 0.001
interquartile range	(−0.2–1.7)	(−1.0–0.6)	

BMI—Body mass index; FAOD—Fatty acid oxidation disorder; SD—standard deviation.

**Table 2:** Characteristics of the studies with control groups.

Author	Year	Country	Study Design	Number of Participants	Types of FAOD	Age (Mean Range)	Sex (%)
de Castro et al. [23]	2021	Spain	observational	FAOD = 10 Control = 20	MCADD = 6 SCADD = 4	5–19 ^1^ Control NI	FAOD F = 70% M = 30% Control NI
Knottnerus et al. [24]	2020	Netherlands	observational	FAOD = 14 Control = 14	VLCADD = 8 LCHADD = 2 CPT2D = 4	FAOD 41 (18–57) Control 38 (18–60)	FAOD F = 21% M = 79% Control F = 21% M = 79%
Madsen et al. [17]	2019	Denmark	observational	FAOD = 2 Control = 4	LCHADD = 2	FAOD 20.5 (15–26) Control 24.8 (19–30)	FAOD F = 50% M = 50% Control F = 75% M = 25%
Madsen et al. [25]	2019	Denmark	observational	FAOD = 2 Control = 10	MADD = 2	FAOD 35 (20–50) Control 32 (18–65)	FAOD F = 100% Control F = 70% M = 30%
McCoin et al. ^(s)^ [15]	2019	USA	observational	FAOD = 12 Control = 12	CPT2D = 2 LCHAD = 10	FAOD 14.7 (7–37) Control 15.3 (9–34)	FAOD F = 42% M = 58% Control F = 42% M = 58%
McCoin et al. ^(s)^ [14]	2016	USA	observational	FAOD = 12 Control = 11	CPT2D = 2 LCHAD = 10	FAOD 28 (13–37) Control 26 (NI)	FAOD F = 42% M = 58% Control F = 45% M = 55%
Diekman et al. [18]	2015	Netherlands	observational	FAOD = 5 Control = 5	VLCADD = 5	FAOD 14.7 (NI) Control 15.7 (NI)	FAOD F = 42% M = 58% Control F = 45% M = 55%
Gillingham et al. ^(s)^ [16]	2013	USA	observational	FAOD = 9 Control = 9	LCHADD = 9	FAOD 12.7 (7–17) Control 13.7 (8–22)	FAOD F = 33% M = 67% Control F = 33% M = 67%
Huidekopper et al. [19]	2013	Netherlands	observational	FAOD = 4 Control = 4	MCADD = 4	FAOD 27.3 (21–41) Control 27 (21–32)	FAOD F = 25% M = 75% Control F = 25% M = 75%
Akamizu et al. [20]	2012	Japan	observational	FAOD = 4 Control = 20	CPT2D = 1 MCADD = 2 VLCADD = 1	FAOD 8 (5–11) Control 32.6 (NI)	FAOD F = 100% Control NI
Fletcher et al. [21]	2001	Australia	observational	FAOD = 3 Control = 6	MCADD = 3	FAOD 2.7 (0.9–6) Control 3.7 (3.1–4.3)	FAOD F = 67% M = 33% Control NI
Jakobs et al. [22]	1997	Netherlands	observational	FAOD = 3 Control = 6	CPT1D = 1 MCADD = 1 MADD = 1	FAOD 5.4 (2.6–9) Control 14.3 (2.1–34.8)	FAOD F = 33% M = 67% Control F = 50% M = 50%

^1^ Age range given for the whole study group (FAOD, aminoacidopathies, organic acidaemias). ^(s)^ Studies by McCoin et al., 2019 [15], Gilingham et al., 2013 [16] have overlapping patient/control groups. Studies were performed in the same centres. CPT2D—Carnitine palmitoyltransferase II (CPT II) deficiency; F—Female, FAOD—Fatty acid oxidation disorders; LCHADD—Long-chain 3-hydroxyacyl-CoA dehydrogenase deficiency; M—Male, MADD—Multiple acyl-CoA dehydrogenase deficiency; MCADD—Medium chain acyl-CoA dehydrogenase deficiency, NI—no information; SCADD—Short chain acyl-CoA dehydrogenase deficiency, TFPD—Trifunctional protein deficiency; VLCADD—Very long chain acyl-CoA dehydrogenase deficiency.

**Table 3:** Data extracted form studies with control groups.

Study	No of Patients/Controls	Weight [kg]					BMI [kg/m ^2^]					
		Mean	SD	Median	Z-Score Median	Z-Score SD	Mean	SD	Median	Z-Score Median	Z-Score SD	Percentile Median
de Castro et al., 2021 [23]	FAOD = 10	-	-	-	0.9	1.0	-	-	-	0.6	1.3	-
	CG = 20	-	-	-	0.3	0.9	-	-	-	−0.7	1.0	-
Knottnerus et al., 2020 [24]	FAOD = 14	-	-	-	-	-	*25.3* ^1^	*4.0* ^1^	*25.4* ^1^	-	-	-
	CG = 14	-	-	-	-	-	-	-	25	-	-	-
McCoin el.al., 2019 [15]	LCHAD = 10	-	-	-	-	-	*22.8* ^1^	*4.1* ^1^	*23.2* ^1^	-	-	-
	CPT2 = 2	-	-	-	-	-	*26.3* ^1^	*5.1* ^1^	*26.3* ^1^	-	-	-
	CG = 12	-	-	-	-	-	*22.4* ^1^	*4.9* ^1^	*21.7* ^1^	-	-	-
Madsen et al., 2019 [25]	FAOD = 2	-	-	-	-	-	*29* ^1^	*6.4* ^1^	*29* ^1^	-	-	-
	CG = 10	-	-	-	-	-	32	14	-	-	-	-
Madsen et al., 2019 [17]	FAOD = 2	-	-	-	-	-	*20* ^1^	*2.8* ^1^	*20* ^1^	-	-	-
	CG = 4	-	-	-	-	-	*23* ^1^	*4. 5* ^1^	*24* ^1^	-	-	-
Diekman et al., 2016 [18]	FAOD = 5	*76* ^1^	*14* ^1^	*79* ^1^	-	-	*24.8* ^1^	*4.8* ^1^	*24.9* ^1^	-	-	-
	CG = 5	71	*16* ^1^	-	-	-	21.8	*2.9* ^1^	-	-	-	-
McCoin et al., 2016 ^(s)^ [14]	LCHAD = 10	55.9	*16.4* ^1^	-	-	-	22.8	*4.1* ^1^	-	1.1	-	84.6
	CPT2 = 2	73.4	*17.5* ^1^	-	-	-	26.3	*5.1* ^1^	-	0.5 ^2^	-	70 ^2^
	FAOD = 12	*58.8* ^1^	*17.2* ^1^	-	-	-	*23.4* ^1^	*4.2* ^1^	*-*	*1.0*	*-*	*83.3* ^1^
	CG = 11	61	*21.2* ^1^	-	-	-	23.1	*4.6* ^1^	-	0.7 ^3^	-	72.3 ^3^
Gillingham et al., 2013 ^(s)^ [16]	FAOD = 9	*55.7* ^1^	*17.4* ^1^	*64.7* ^1^	*-*	*-*	*22.3* ^1^	*4.0* ^1^	*22.6* ^1^	*1* ^1^	*-*	*83* ^1^
	CG = 9	*55.0* ^1^	*24.4* ^1^	*48.9* ^1^	*-*	*-*	*21.4* ^1^	*5.1* ^1^	*19.7* ^1^	*0.4* ^1^	*-*	*63* ^1^
Huidekopper et al., 2013 [19]	FAOD = 4	*81.3* ^1^	*15.05* ^1^	*79.7* ^1^	*-*	*-*	*24.5* ^1^	*3.8* ^1^	*24.1* ^1^	*-*	*-*	-
Akamizu et al., 2012 [20]	FAOD = 4	*-*	*-*	*-*	*-*	*-*	*15.9* ^1^	*1.4* ^1^	*15.6* ^1^	*-*	*-*	*-*
	CG = 20	-	-	-	-	-	20.3	1.9	-	-	-	-
Fletcher et al., 2001 [21]	MCADD = 3	*22.5* ^1^	*7.0* ^1^	*11.7* ^1^	-	-	-	-	-	-	-	-
	CG = 6	*15.3* ^1^	*1.0* ^1^	*15.8* ^1^	*-*	-	-	-	-	-	-	-
Jakobs et al., 1997 [22]	FAOD = 3	*21.5* ^1^	*10.5* ^1^	*19* ^1^	-	-	-	-	-	-	-	-
	CG = 6	*31.9* ^1^	*23.6* ^1^	*22.3* ^1^	-	-	-	-	-	-	-	-

^1^ Data in italics were calculated based on individual patient data; ^2^ Data from 1 patient; ^3^ Data from 9 patients; ^(s)^ Studies by McCoin et al., 2019 [15], Gilingham et al., 2013 [16] have overlapping patient/control groups. Studies were performed in the same centres [14,15,16]. CG—control group, CPT2D—Carnitine palmitoyltransferase II (CPT II) deficiency; FAOD—Fatty acid oxidation disorders; LCHADD—Long-chain 3-hydroxyacyl-CoA dehydrogenase deficiency.

**Table 4:** Certainty of evidence evaluated using GRADE framework.

Outcome	Group	No of Studies	Patients	Controls	MD (95% CI)	*p*-Value	I^2^	Risk of Bias	Inconsistency	Indirections	Imprecision	Grade
Weight	FAOD	6	63	186	1.23 (−2.55, 5.00)	0.53	0	Downgrade 1 level	No downgrade	Downgrade 1 level	No downgrade	⨁◯◯◯ Very low
	MCADD	3	46	166	−0.51 (−5.02, 3.88)	0.97	0	Downgrade 1 level	No downgrade	No downgrade	No downgrade	⨁◯◯◯ Very low
	Other FAOD	3	56	176	6.3 (−0.94, 12.49)	0.09	0	Downgrade 1 level	No downgrade	Downgrade 1 level	Downgrade 1 level	⨁◯◯◯ Very low
BMI	FAOD	7	68	216	0.26 (−2.33, 2.85)	0.84	71.05	Downgrade 1 level	Downgrade 1 level	Downgrade 1 level	Downgrade 1 level	⨁◯◯◯ Very low
	MCADD	3	47	180	−1.1 (−5.21, 3.09)	0.62	83.6	Downgrade 1 level	Downgrade 2 levels	No downgrade	Downgrade 1 level	⨁◯◯◯ Very low
	Other FAOD	6	64	212	0.70 (−1.90, 3.26)	0.60	57.2	Downgrade 1 level	Downgrade 1 level	No downgrade	Downgrade 1 level	⨁◯◯◯ Very low

95% CI—95% Confidence Interval; FAOD—Fatty acid oxidation disorders; MCADD—Medium-chain acyl-CoA dehydrogenase deficiency; MD—difference in means, ⨁◯◯◯—very low quality of evidence.

## Data Availability

The data presented in this article are available upon request from the corresponding author.

## Supplementary Materials

The following supporting information can be downloaded at: https://www.mdpi.com/article/10.3390/healthcare10122405/s1, Table S1. Characteristics of the studies without control groups, Table S2. Data extracted form studies without control groups, Table S3: PRISMA 2020 Checklist; Figure S1: Funnel plot of Standard Error by a difference in the means of weight (FAOD vs. Controls); Figure S2: Funnel plot of Standard Error by a difference in the means of BMI (FAOD vs. Controls); Figure S3: Funnel plot of Standard Error by a difference in the means of weight (MCADD vs. Controls); Figure S4: Funnel plot of Standard Error by a difference in the means of BMI (MCADD vs. Controls) Figure S5: Funnel plot of Standard Error by a difference in the means of weight (other FAOD vs. Controls); Figure S6: Funnel plot of Standard Error by a difference in the means (other FAOD vs. Controls); Figure S7: Cumulative meta-analysis: Forest plot: comparison of body weight in FAOD patients vs. controls; Figure S8: Sensitivity meta-analysis: Forest plot: comparison of weight in FAOD patients vs. controls; Figure S9: Cumulative meta-analysis: Forest plot: comparison of BMI in FAOD patients vs. controls; Figure S10: Sensitivity meta-analysis: Forest plot: comparison of BMI in FAOD patients vs. controls; Figure S11: Cumulative meta-analysis: Forest plot: comparison of weight in MCADD patients vs. controls; Figure S12: Sensitivity meta-analysis: Forest plot: comparison of weight in MCADD patients vs. controls; Figure S13: Cumulative meta-analysis: Forest plot: comparison of BMI in MCADD patients vs. controls; Figure S14: Sensitivity meta-analysis: Forest plot: comparison of BMI in MCADD patients vs. controls; Figure S15: Cumulative meta-analysis: Forest plot: comparison of weight in patients with types of FAOD other than MCADD vs. controls; Figure S16: Sensitivity meta-analysis: Forest plot: comparison of weight in patients with types of FAOD other than MCADD vs. controls; Figure S17: Cumulative meta-analysis: Forest plot: comparison of BMI in patients with types of FAOD other than MCADD vs. controls; Figure S18: Sensitivity meta-analysis: Forest plot: comparison of BMI in patients with types of FAOD other than MCADD vs. controls.
