# Supplementary material for: Anthropometric Parameters in Patients with Fatty Acid Oxidation Disorders: A Case–Control Study, Systematic Review and Meta-Analysis
Source: Healthcare (Basel). 2022 Nov 30;10(12):2405. doi: 10.3390/healthcare10122405 (PMC9777909; doi:10.3390/healthcare10122405)
Supplement: Supplementary file 1 [file healthcare-10-02405-s001.zip › healthcare-2028260-supplementary.pdf]

healthcare

MDPI

Supplementary Materials

Table S1. Characteristics of studies without control groups.

Author	Year	Country	Study design	Number of participants	Types of FAOD	Age (mean (range)	Sex (%)
Schwantje.t al.[1] [1]	2022	Netherlands	observational	FAOD = 8 (of 13)	LCHADD = 7<br>TFPD = 1	9.43 (3.9–13.3)	F = 50%<br>M = 50%
Schwantje et al.[2] [2]	2022	Netherlands	observational	FAOD = 5	TFPD = 5	17.2 (12–24)	F = 40%<br>M = 60%
Storgaard et al.[3] [3]	2022	Denmark	experimental	FAOD = 8	CPT2D = 4<br>VLCADD = 4	39.6 (18–80)	F = 37.5%<br>M = 62.5%
Norris et al. [4]	2021	USA	observational	FAOD = 3	VLCADD = 2<br>CACTD = 1	4.0 (2.25–5)	F = 33%<br>M = 67%
Rücklova et al. [5]	2021	Czech Republic	observational	FAOD = 97 (3)	LCHAD = 28<br>MCADD = 69	NI (0.1–36.6)	F = 49%<br>M = 51%
Guffon et al. [6]	2021	France	observational	FAOD = 18	LCHADD = 5<br>VLCADD = 5<br>CPT2D = 3<br>CATCD = 3<br>TFPD = 2	NI	F = 44%<br>M = 56%
Stenlid et al. [7].	2021	Sweden	observational	FAOD = 21	VLCADD = 9<br>MCADD = 7<br>CUD = 5	6.4 (NI)	F = 33%<br>M = 67%
Zöggeler et al. [8]	2021	Austria	observational	FAOD = 12	LCHADD = 9<br>CPT2D = 1<br>VLCADD = 2	9.1 (3.1–32)	F = 50%<br>M = 50%
Vockley et al [9,10]<br>Lee et al, 2021[4] [11]	2019<br>2017	USA	observational	FAOD = 29	LCHADD = 10<br>CPT2D = 4<br>VLCADD = 12<br>TFPD = 3	12.1 (0.9–58.8)	F = 41%<br>M = 59%
Anderson et al. [12]	2019	USA	observational	FAOD = 90	MCADD = 90	9.1 (1.6–28.5)	F = 49%<br>M = 51%
Gillingham et al. [(s)] [13]	2019	USA	experimental,	FAOD = 13	LCHADD = 10<br>CPT2D = 2<br>VLCADD = 1	15.5 (7–37)	F = 46%<br>M = 54%
Rovelli et al. [14]	2019	USA	observational	FAOD = 26	VLCADD = 26	7.0 (0.6–22.9)	F = 65%<br>M = 35%

Shiraishi et al.[5] [15]	2019	Japan	experimental	FAOD = 6	CPT2D = 1<br>VLCADD = 5	15 (6–26)	F = 67%<br>M = 33%
Yamada et al.[5] [16]	2018	Japan	experimental	FAOD = 8	CPT2D = 2<br>VLCADD = 6	13.25 (6–26)	F = 75%<br>M = 25%
Bleeker et al. [17]	2018	Netherlands	observational	FAOD = 16	VLCADD = 16	19.5 (13–45)	F = 44%<br>M = 56%
MacDonald et al.[6] [18]	2018	UK	experimental	FAOD = 5	VLCADD = 2<br>CATCD = 2<br>LCHADD = 1	8.1 (7–13)	F = 40%<br>M = 60%
Gillingham et al. [(s)] [19]	2017	USA	experimental	FAOD = 32	LCHADD/TFP = 12<br>CPT2D = 11<br>VLCADD = 9	25.03 (7–64)	F = 62.5%<br>M = 37.5%
De Biase et al.[(s)] [20]	2016	USA	observational	FAOD = 5	LCHADD = 4<br>TFPD = 1	11.6 (2–22)	F = 40%<br>M = 60%
Evans et al.[7] [21]	2016	Australia	observational	FAOD = 22	VLCADD = 22	NI	NI
Haglind et al. [22]	2015	Sweden	observational	FAOD = 5	LCHADD = 5	7.5 (5.5–9.5)	F = 80%<br>M = 20%
Ørngreen et al. [23]	2014	Denmark/ France	experimental	FAOD = 10	CPT2D = 5<br>VLCADD = 5	39 (16–65)	F = 20%<br>M = 80%
Behrend et al.[(s)] [24]	2012	USA	experimental	FAOD = 11	LCHADD = 8<br>CPT2D = 2<br>VLCADD = 1	16.5 (7–37)	F = 45%<br>M = 55%
Haglind et al. [22]	2012	Sweden	observational	FAOD = 10	LCHADD = 10	NI	NI
Gillingham et al. [25]	2007	USA	experimental	FAOD = 9	LCHADD = 8<br>TFPD = 1	11.4 (7.5–14.4)	F = 67%<br>M = 33%
Lafolla [26]	1994	USA	observational	FAOD = 120	MCADD = 120	NI (0–20)	F = 55%<br>M = 45%

[1] Study reports 13 patients, 5 of whom died in infancy. Here, we report only data from living patients.
[2]Study reports anthropometric data for 4 out of 5 patients. One of the patients was included in both studies by Schwantje.
[3]Anthropometric data was available from 26 out of 87 patients
[4]Studies by Vockley 2017, 2019 describe the same patient group (the same trial). Study by Lee uses data from Vockley 2017
[5] Studies by Yamada and Shiraishi describe the same patient group (the same trial). However, two patients were drop-outs.
[6] 1 patient dropped out and her data was not shown
[7] Data from 16 patients for BMI, from 9 patients for body composition, out of 22
[(s)] Studies by McCoin et al. 2019, 2016, Gilingham et al. 2013,2017,2019, de Biase et al. 2016 and Behrend et al. 2012 have overlapping patient/control groups; Studies were performed in the same centers.

CATCD - Carnitine-acylcarnitine translocase deficiency; CPT2D - Carnitine

palmitoyltransferase II (CPT II) deficiency; CUD - carnitine uptake deficiency, F – Female, FAOD - Fatty acid oxidation disorders; LCKATD - long-chain 3-ketothiolase deficiency; LCHADD - Long-chain 3-hydroxyacyl-CoA dehydrogenase deficiency; M – Male, MCADD - Medium chain acyl-CoA dehydrogenase deficiency, NI - no information; TFPD - Trifunctional protein deficiency; VLCADD - Very long-chain acyl-CoA dehydrogenase deficiency

Table S2. Data extracted form studies without control groups.

		**Weight [kg]**				**BMI [kg/m²]**				
**Study**	**No of patients / controls**	**Mean**	**SD**	**Median**	**Z-score SD**	**Mean**	**SD**	**Median**	**Z-score SD**	**BMI percentile median**
Storgaard et al., 2022[2] [3]	8	-	-	-	-	*28.9*[1]	*7.2*[1]	*29*[1]	-	-
Schwantje et al., 2022[3] [2]	4	-	-	-	*0.2*[1]	-	-	-	-	-
Norris et al., 2021 [4]	3	*18.3*[1]	*5.9*[1]	*18.5*[1]	-	-	-	-	-	-
Stenlid et al., 2021 [7]	21	*26.9*[1]	-	-	-	*18.2*[1]	-	-	-	-
Zöggeler et al., 2021 [8]	12	*39.6*[1]	*24.5*[1]	*34.3*[1]	-	*18.6*[1]	*4.3*[1]	*17.9*[1]	-	*55*[1]
Gillingham et al., 2019[(s)] [13]	13	*62.2*[1]	*18.4*[1]	*65*[1]	-	*24*[1]	*4.7*[1]	*24*[1]	-	-
Vockley et al., 2017, 2019 [9,10]	29	42.2	34.1	228	-	-	-	-	-	-
Shiriashi et al., 2019 [15]	6	*36.5*[1]	*19.2*[1]	*34*[1]	-	-	-	-	-	-
Yamada et al., 2018 [16]	8	*34.8*[1]	*16.8*[1]	*29.5*[1]	-	-	-	-	-	-
Bleeker et al., 2018 [17]	16	*78.2*[1]	*14.0*[1]	*79.7*[1]	*1.8*[1]	*26.1*[1]	*4.5*[1]	*25.3*[1]	*1.8*[1]	-
Gillingham et al., 2017[(s)] [19]	32	*64.6*[1]	*24.9*[1]	*65*[1]	-	*24.1*[1]	*5.5*[1]	*23.8*[1]	-	-
MacDonald et al., 2017 [18]	5	*51.8*[1]	*13.2*[1]	*51*[1]	-	*24.8*[1]	*3.4*[1]	*26.5*[1]	-	-
Evans et al., 2016 [21]	23	-	-	-	-	-	-	-	*−0.11*[1,4]	-

Haglind et al., 2015 [27]	5	-	-	-	*1.3*[1]	-	-	-	*1.6*[1]	-
Ørngreen et al., 2014 [23]	B = 4[5]<br>P = 6[6]	-	-	-	-	*B = 30*[1,5]<br>*P=25*[1,6]	*1*[1]<br>*1*[1]	-	-	-
Behrend et al., 2012[(s)] [24]	11	-	-	-	-	*23.6*[1]	*4.9*[1]	*22.7*[1]	-	-
Gillingham et al., 2007 [25]	9	-	-	-	-	18.7	4.4	18.9	-	50

[1]Data in italics was calculated based on individual patient data
[2]Data from 8 out of 13 patients was included into the analysis. 5 patients died in early infancy.
[3]Data from 4 out of 5 patients. Missing data for 9 patients.
[4] BMI z-scores were available for 9 patients only
[5] Data from patients in bezafibrate group
[6]Data from patients in the placebo group
[(s)] Studies by McCoin et al. 2019, 2016, Gilingham et al. 2013,2017,2019, de Biase et al. 2016 and Behrend et al. 2012 have overlapping patient/control groups; Studies were performed in the same centers.

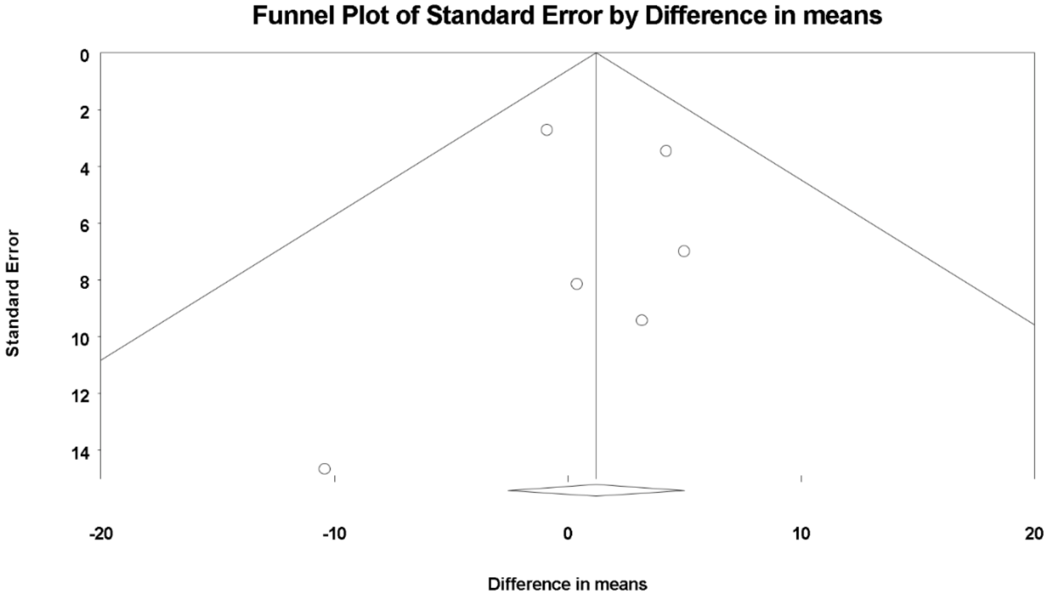

Figure S1. Funnel plot of Standard Error by a difference in Means of weight (FAOD vs. Controls).

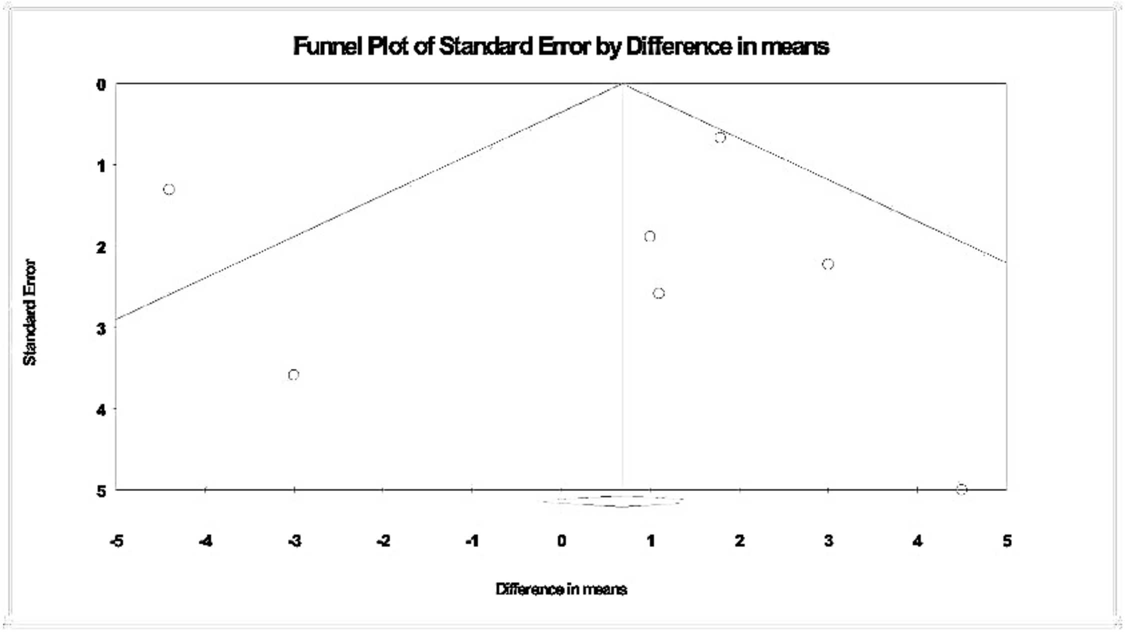

Figure S2. Funnel plot of Standard Error by a difference in Means of BMI (FAOD vs. Controls).

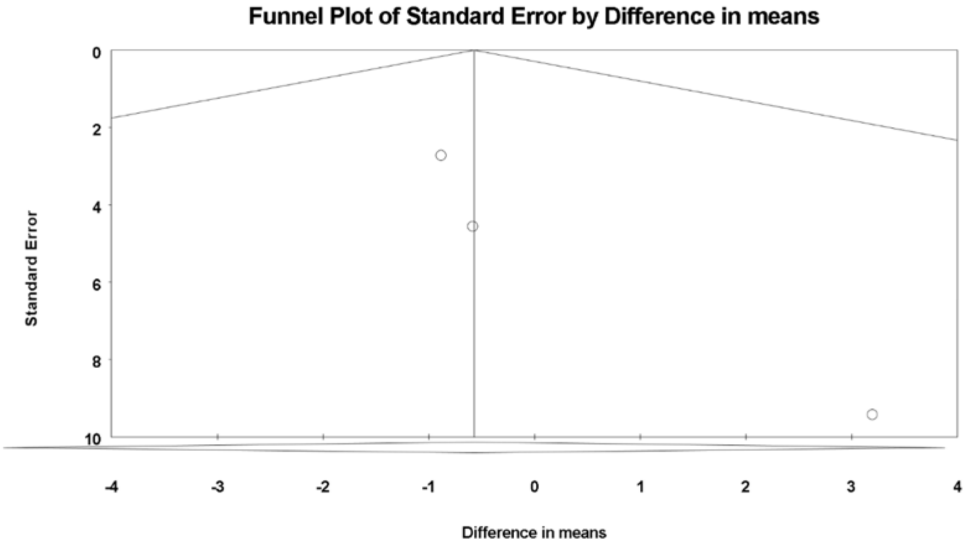

Figure S3. Funnel plot of Standard Error by a difference in Means of weight (MCADD vs. Controls).

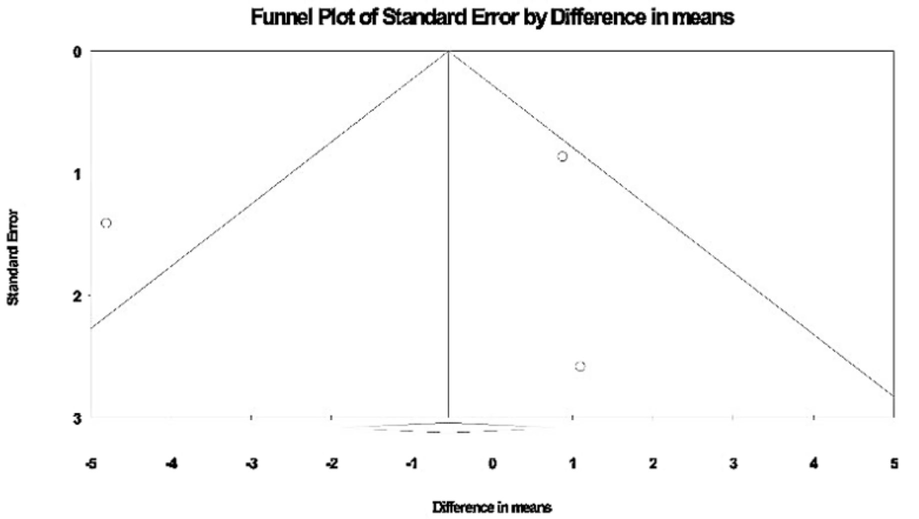

Figure S4. Funnel plot of Standard Error by a difference in Means of BMI (MCADD vs. Controls).

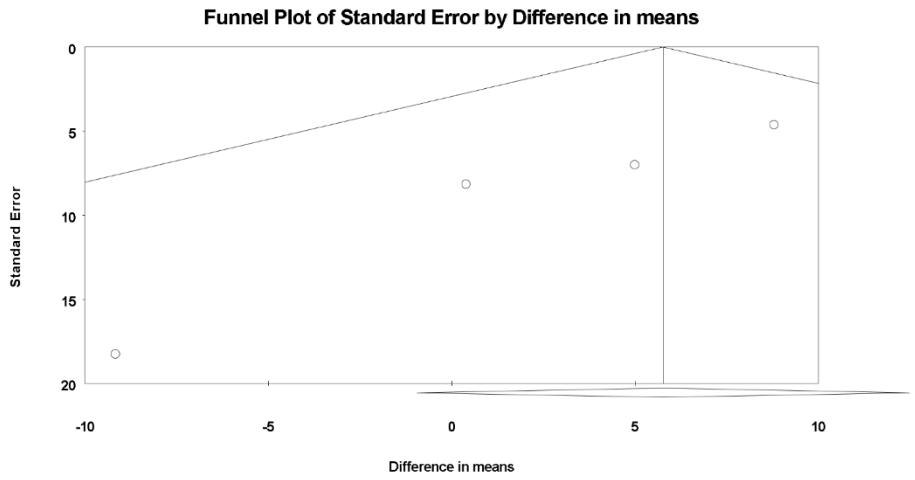

Figure S5. Funnel plot of Standard Error by a difference in Means of weight (other FAOD vs. Controls).

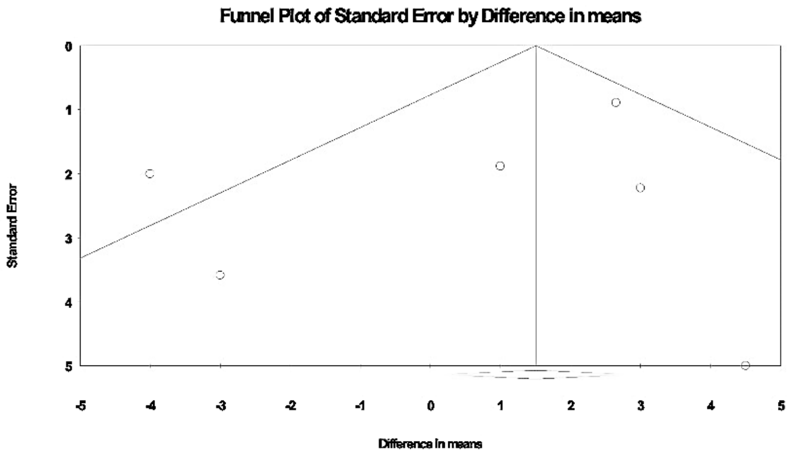

Figure S6. Funnel plot of Standard Error by a difference in Means (other FAOD vs. Controls).

Study name	Cumulative statistics									Relative weight	Relative weight
	Point	Standard error	Variance	Lower limit	Upper limit	Z-Value	p-Value	FAOD	Control		
Jakobs, 1997	-10,399	14,663	214,991	-39,137	18,339	-0,709	0,478	3	6	1,73	
Fletcher, 2001	-1,198	2,679	7,177	-6,449	4,053	-0,447	0,655	6	12	51,72	
Gillingam, 2013	-1,042	2,545	6,478	-6,031	3,946	-0,409	0,682	15	21	57,30	
Huidekopper, 2013	-0,754	2,457	6,038	-5,570	4,062	-0,307	0,759	19	25	61,47	
Diekman, 2015	-0,123	2,319	5,376	-4,667	4,422	-0,053	0,958	24	30	69,05	
Wasiewicz-Gajdzis, 2022	1,225	1,927	3,712	-2,551	5,001	0,636	0,525	63	186	100,00	
	1,225	1,927	3,712	-2,551	5,001	0,636	0,525				

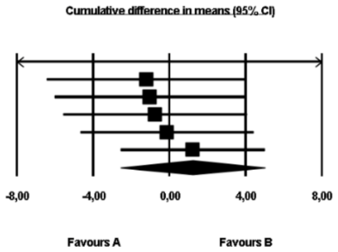

Figure S7. Cumulative meta-analysis - Forest plot - comparison of body weight in FAOD patients vs. controls [28–32].

Study name	Statistics with study removed						
	Point	Standard error	Variance	Lower limit	Upper limit	Z-Value	p-Value
Wasiewicz-Gajdzis, 2022	3,085	4,628	21,418	-5,986	12,156	0,667	0,505
Gillingam, 2013	4,266	2,948	8,692	-1,512	10,044	1,447	0,148
Huidekopper, 2013	3,878	2,901	8,415	-1,808	9,563	1,337	0,181
Diekman, 2015	3,599	3,020	9,118	-2,319	9,518	1,192	0,233
	3,819	2,773	7,687	-1,615	9,253	1,377	0,168

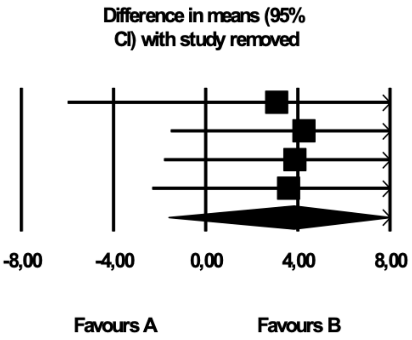

Figure S8. – Sensitivity meta-analysis - Forest plot - comparison of weight in FAOD patients vs. controls [30–32].

Study name	Cumulative statistics									Relative weight	Relative weight
	Point	Standard error	Variance	Lower limit	Upper limit	Z-Value	p-Value	FAOD	Control		
Akamizu, 2012	-4,400	1,305	1,702	-6,957	-1,843	-3,373	0,001	4	2	19,65	
Huidekopper, 2013	-2,102	2,713	7,358	-7,419	3,215	-0,775	0,438	8	6	32,26	
Diekman, 2015	-0,389	2,570	6,606	-5,427	4,649	-0,151	0,880	13	11	46,67	
McCoin, 2019 & 2016	-0,110	1,911	3,653	-3,856	3,636	-0,057	0,954	25	23	62,95	
Madsen, 2019a	0,308	1,802	3,248	-3,224	3,840	0,171	0,864	27	25	68,39	
Madsen, 2019b	-0,146	1,588	2,523	-3,259	2,967	-0,092	0,927	29	29	77,12	
Wasiewicz-Gajdzis, 2022	0,262	1,323	1,751	-2,332	2,855	0,198	0,843	68	185	100,00	
	0,262	1,323	1,751	-2,332	2,855	0,198	0,843				

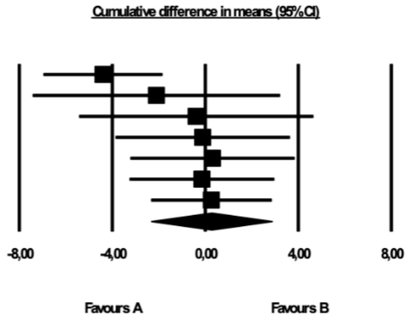

Figure S9. Cumulative meta-analysis - Forest plot - comparison of BMI in FAOD patients vs. controls [31–36].

Study name	Statistics with study removed						
	Point	Standard error	Variance	Lower limit	Upper limit	Z-Value	p-Value
Wasiewicz-Gajdzis, 2022	-0,146	1,588	2,523	-3,259	2,967	-0,092	0,927
McCoin, 2019 & 2016	0,150	1,592	2,534	-2,970	3,270	0,094	0,925
Madsen, 2019a	0,017	1,388	1,926	-2,703	2,737	0,012	0,990
Madsen, 2019b	0,584	1,410	1,988	-2,179	3,348	0,414	0,679
Diekman, 2015	-0,189	1,501	2,254	-3,132	2,753	-0,126	0,900
Huidekopper, 2013	0,156	1,505	2,266	-2,794	3,106	0,104	0,917
Akamizu, 2012	1,675	0,576	0,332	0,546	2,804	2,908	0,004
	0,262	1,323	1,751	-2,332	2,855	0,198	0,843

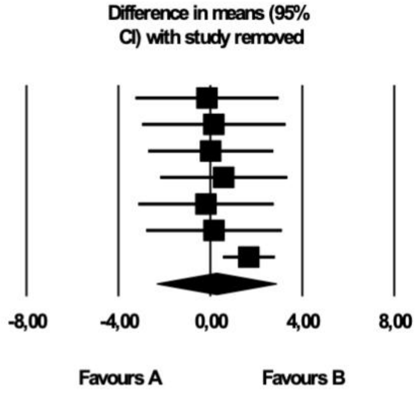

Figure S10. Sensitivity meta-analysis - Forest plot - comparison of BMI in FAOD patients vs. controls [31–37].

Study name	Point	Standard error	Variance	Lower limit	Upper limit	Z-Value	p-Value	MCAD	Control	Relative weight	Relative weight
	Cumulative statistics										
Fletcher, 2001	-0,880	2,725	7,425	-6,221	4,461	-0,323	0,747	3	6	69,44	
Huidekopper, 2013	-0,566	2,618	6,853	-5,696	4,565	-0,216	0,829	7	10	75,24	
Wasiewicz-Gajdzis, 2022	-0,569	2,271	5,156	-5,020	3,881	-0,251	0,802	26	166	100,00	
	-0,569	2,271	5,156	-5,020	3,881	-0,251	0,802				

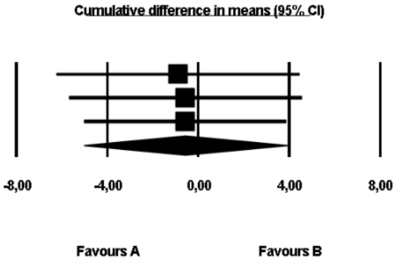

Figure S11. – Cumulative meta-analysis - Forest plot - comparison of weight in MCADD patients vs. controls [29,31].

Study name	Point	Standard error	Variance	Lower limit	Upper limit	Z-Value	p-Value
	Statistics with study removed						
Wasiewicz-Gajdzis, 2022	3,200	9,430	88,925	-15,282	21,682	0,339	0,734
Huidekopper, 2013	-0,580	4,563	20,821	-9,523	8,363	-0,127	0,899
	0,137	4,107	16,871	-7,913	8,187	0,033	0,973

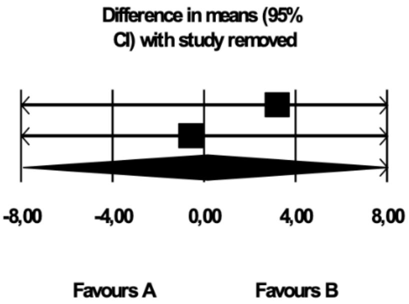

Figure S12. Sensitivity meta-analysis - Forest plot - comparison of weight in MCADD patients vs. controls [31].

Study name	Point	Standard error	Variance	Lower limit	Upper limit	Z-Value	p-Value	MCAD	Control	Relative weight	Relative weight
	Cumulative statistics										
Akamizu, 2012	-4,800	1,409	1,985	-7,561	-2,039	-3,407	0,001	2	2	35,23	
Huidekopper, 2013	-2,247	2,923	8,545	-7,976	3,482	-0,769	0,442	6	6	60,98	
Wasiewicz-Gajdzis, 2022	-1,065	2,117	4,482	-5,214	3,085	-0,503	0,615	25	162	100,00	
	-1,065	2,117	4,482	-5,214	3,085	-0,503	0,615				

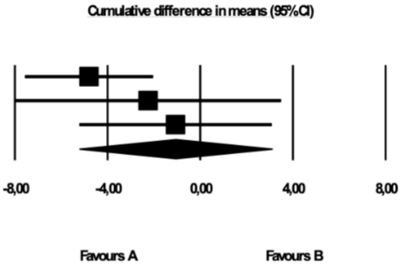

Figure S13. – Cumulative meta-analysis - Forest plot - comparison of BMI in MCADD patients vs. controls [31,33].

Study name	Point	Standard error	Variance	Lower limit	Upper limit	Z-Value	p-Value
	Statistics with study removed						
Wasiewicz-Gajdzis, 2022	-2,247	2,923	8,545	-7,976	3,482	-0,769	0,442
Huidekopper, 2013	-1,851	2,838	8,054	-7,414	3,711	-0,652	0,514
Akamizu, 2012	0,902	0,821	0,675	-0,708	2,512	1,099	0,272
	-1,065	2,117	4,482	-5,214	3,085	-0,503	0,615

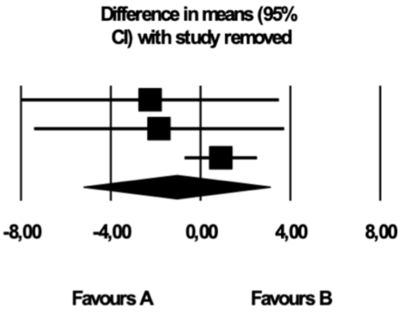

Figure S14. – Sensitivity meta-analysis - Forest plot - comparison of BMI in MCADD patients vs. controls [31,33].

Study name	Cumulative statistics										Relative weight	Relative weight
	Point	Standard error	Variance	Lower limit	Upper limit	Z-Value	p-Value	Other FAOD	Control			
Jakobs, 1997	-9,167	18,260	333,416	-44,955	26,621	-0,502	0,616	2	6		3,52	
Gillingam, 2013	-1,191	7,446	55,450	-15,786	13,404	-0,160	0,873	11	15		21,18	
Diekman, 2015	2,096	5,100	26,013	-7,901	12,092	0,411	0,681	16	20		45,15	
Wasiewicz-Gajdzis, 2022	5,773	3,427	11,744	-0,944	12,490	1,685	0,092	36	176		100,00	
	5,773	3,427	11,744	-0,944	12,490	1,685	0,092					

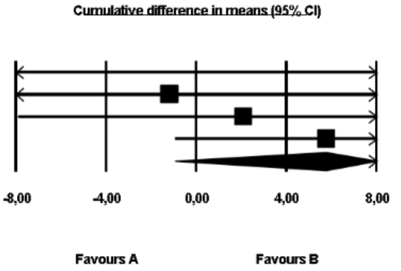

Figure S15. Cumulative meta-analysis - Forest plot - comparison of weight in patients with types of FAOD other than MCADD vs. controls [28,30,32].

Study name	Statistics with study removed						
	Point	Standard error	Variance	Lower limit	Upper limit	Z-Value	p-Value
Wasiewicz-Gajdzis, 2022	3,049	5,312	28,214	-7,362	13,459	0,574	0,566
Diekman, 2015	6,754	4,024	16,196	-1,133	14,642	1,678	0,093
Gillingam, 2013	7,645	3,860	14,900	0,079	15,210	1,980	0,048
	6,319	3,489	12,173	-0,520	13,157	1,811	0,070

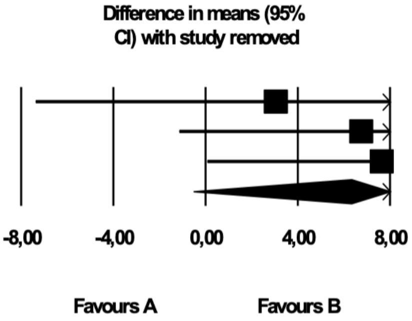

Figure S16. – Sensitivity meta-analysis - Forest plot - comparison of weight in patients with types of FAOD other than MCADD vs. controls [30,32].

Study name	Cumulative statistics									Relative weight	Relative weight
	Point	Standard error	Variance	Lower limit	Upper limit	Z-Value	p-Value	Other FAOD	Control		
Akamizu, 2012	-1,998	1,224	1,499	-4,397	0,402	-1,632	0,103	2	2	5,19	
Diekman, 2015	-0,386	1,413	1,997	-3,155	2,384	-0,273	0,785	7	7	19,55	
McCoin, 2019 & 2016	0,075	0,575	0,331	-1,052	1,202	0,130	0,897	19	19	45,49	
Madsen, 2019a	0,244	0,453	0,205	-0,644	1,132	0,539	0,590	21	21	52,31	
Madsen, 2019b	0,099	0,401	0,161	-0,686	0,884	0,247	0,805	23	25	61,32	
Wasiewicz-Gajdzis, 2022	0,345	0,294	0,086	-0,232	0,921	1,172	0,241	43	181	100,00	
	0,345	0,294	0,086	-0,232	0,921	1,172	0,241				

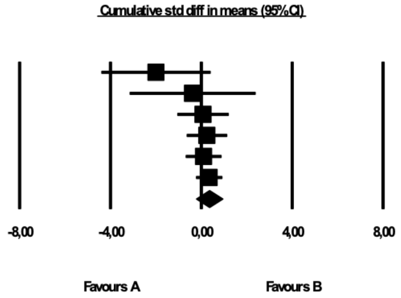

Figure S17. Cumulative meta-analysis - Forest plot - comparison of BMI in patients with types of FAOD other than MCADD vs. controls [32–37].

Study name	Statistics with study removed						
	Point	Standard error	Variance	Lower limit	Upper limit	Z-Value	p-Value
Wasiewicz-Gajdzis, 2022	-0,094	1,597	2,551	-3,225	3,036	-0,059	0,953
McCoin, 2019 & 2016	0,540	1,713	2,935	-2,818	3,898	0,315	0,753
Madsen, 2019a	0,423	1,421	2,018	-2,361	3,208	0,298	0,766
Madsen, 2019b	1,087	1,384	1,914	-1,624	3,799	0,786	0,432
Diekman, 2015	0,150	1,603	2,569	-2,991	3,292	0,094	0,925
Akamizu, 2012	2,242	0,732	0,536	0,807	3,677	3,062	0,002
	0,693	1,321	1,744	-1,895	3,282	0,525	0,600

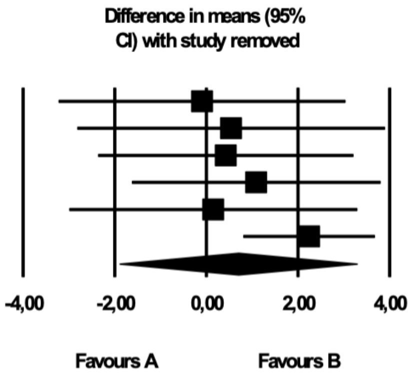

Figure S18. Sensitivity meta-analysis - Forest plot - comparison of BMI in patients with types of FAOD other than MCADD vs. controls [32–37].

Table S3. – PRISMA 2020 Checklist.

Section and Topic	Item #	Checklist item	Location where item is reported
**TITLE**			
Title	1	Identify the report as a systematic review.	Page 1
**ABSTRACT**			
Abstract	2	See the PRISMA 2020 for Abstracts checklist.	Page 1
**INTRODUCTION**			
Rationale	3	Describe the rationale for the review in the context of existing knowledge.	Page 2
Objectives	4	Provide an explicit statement of the objective(s) or question(s) the review addresses.	Page 2
**METHODS**			
Eligibility criteria	5	Specify the inclusion and exclusion criteria for the review and how studies were grouped for the syntheses.	Page 3–4
Information sources	6	Specify all databases, registers, websites, organisations, reference lists and other sources searched or consulted to identify studies. Specify the date when each source was last searched or consulted.	Page 3
Search strategy	7	Present the full search strategies for all databases, registers and websites, including any filters and limits used.	Page 3–4
Selection process	8	Specify the methods used to decide whether a study met the inclusion criteria of the review, including how many reviewers screened each record and each report retrieved, whether they worked independently, and if applicable, details of automation tools used in the process.	Page 4
Data collection process	9	Specify the methods used to collect data from reports, including how many reviewers collected data from each report, whether they worked independently, any processes for obtaining or confirming data from study investigators, and if applicable, details of automation tools used in the process.	Page 4
Data items	10a	List and define all outcomes for which data were sought. Specify whether all results that were compatible with each outcome domain in each study were sought (e.g., for all measures, time points, analyses), and if not, the methods used to decide which results to collect.	Page 3
	10b	List and define all other variables for which data were sought (e.g., participant and intervention characteristics, funding sources). Describe any assumptions made about any missing or unclear information.	Page 3–4

Section and Topic	Item #	Checklist item	Location where item is reported
Study risk of bias assessment	11	Specify the methods used to assess risk of bias in the included studies, including details of the tool(s) used, how many reviewers assessed each study and whether they worked independently, and if applicable, details of automation tools used in the process.	Page 4
Effect measures	12	Specify for each outcome the effect measure(s) (e.g., risk ratio, mean difference) used in the synthesis or presentation of results.	Page 5
Synthesis methods	13a	Describe the processes used to decide which studies were eligible for each synthesis (e.g., tabulating the study intervention characteristics and comparing against the planned groups for each synthesis (item #5)).	Page 5, Supplementary Materials
	13b	Describe any methods required to prepare the data for presentation or synthesis, such as handling of missing summary statistics, or data conversions.	Page 4–5
	13c	Describe any methods used to tabulate or visually display results of individual studies and syntheses.	Page 5
	13d	Describe any methods used to synthesize results and provide a rationale for the choice(s). If meta-analysis was performed, describe the model(s), method(s) to identify the presence and extent of statistical heterogeneity, and software package(s) used.	Page 5
	13e	Describe any methods used to explore possible causes of heterogeneity among study results (e.g. subgroup analysis, meta-regression).	Page 5
	13f	Describe any sensitivity analyses conducted to assess robustness of the synthesized results.	Page 5
Reporting bias assessment	14	Describe any methods used to assess risk of bias due to missing results in a synthesis (arising from reporting biases).	-
Certainty assessment	15	Describe any methods used to assess certainty (or confidence) in the body of evidence for an outcome.	Page 5
**RESULTS**			
Study selection	16a	Describe the results of the search and selection process, from the number of records identified in the search to the number of studies included in the review, ideally using a flow diagram.	Page 6–7
	16b	Cite studies that might appear to meet the inclusion criteria, but which were excluded, and explain why they were excluded.	Page 6–7
Study characteristics	17	Cite each included study and present its characteristics.	Page 8–13
Risk of bias in studies	18	Present assessments of risk of bias for each included study.	Page 16
Results of individual studies	19	For all outcomes, present, for each study: (a) summary statistics for each group (where appropriate) and (b) an effect estimates and its precision (e.g., confidence/credible interval), ideally using structured tables or plots.	Page 14–19
Results of syntheses	20a	For each synthesis, briefly summarise the characteristics and risk of bias among contributing studies	Page 16
	20b	Present results of all statistical syntheses conducted. If meta-analysis was done, present for each the summary estimate and its precision (e.g., confidence/credible interval) and measures of statistical heterogeneity. If comparing groups, describe the direction of the effect.	Page 14–19

Section and Topic	Item #	Checklist item	Location where item is reported
	20c	Present results of all investigations of possible causes of heterogeneity among study results.	Supplementary Materials Fig. S1-S6
	20d	Present results of all sensitivity analyses conducted to assess the robustness of the synthesized results.	Supplementary Materials
Reporting biases	21	Present assessments of risk of bias due to missing results (arising from reporting biases) for each synthesis assessed.	Page 16
Certainty of evidence	22	Present assessments of certainty (or confidence) in the body of evidence for each outcome assessed.	Supplementary Materials Fig. S1-S6
**DISCUSSION**			
Discussion	23a	Provide a general interpretation of the results in the context of other evidence.	Page 20
	23b	Discuss any limitations of the evidence included in the review.	Page 20
	23c	Discuss any limitations of the review processes used.	Page 20
	23d	Discuss implications of the results for practice, policy, and future research.	Page 20
**OTHER INFORMATION**			
Registration and protocol	24a	Provide registration information for the review, including register name and registration number, or state that the review was not registered.	Page 3
	24b	Indicate where the review protocol can be accessed, or state that a protocol was not prepared.	Page 3
	24c	Describe and explain any amendments to information provided at registration or in the protocol.	-
Support	25	Describe sources of financial or non-financial support for the review, and the role of the funders or sponsors in the review.	Page 21
Competing interests	26	Declare any competing interests of review authors.	Page 21
Availability of data, code and other materials	27	Report which of the following are publicly available and where they can be found: template data collection forms; data extracted from included studies; data used for all analyses; analytic code; any other materials used in the review.	Page 21

1. Schwantje, M.; Fuchs, S.A.; de Boer, L.; Bosch, A.M.; Cuppen, I.; Dekkers, E.; Derks, T.G.J.; Ferdinandusse, S.; Ijlst, L.; Houtkooper, R.H.; et al. Genetic, Biochemical, and Clinical Spectrum of Patients with Mitochondrial Trifunctional Protein Deficiency Identified after the Introduction of Newborn Screening in the Netherlands. *J. Inherit. Metab. Dis.* **2022**, *45*, 804–818, doi:10.1002/jimd.12502.
2. Schwantje, M.; Ebberink, M.S.; Doolaard, M.; Ruiter, J.P.N.; Fuchs, S.A.; Darin, N.; Hedberg-Oldfors, C.; Régal, L.; Donker Kaat, L.; Huidekoper, H.H.; et al. Thermo-sensitive Mitochondrial Trifunctional Protein Deficiency Presenting with Episodic Myopathy. *J. Inherit. Metab. Dis.* **2022**, *45*, 819–831, doi:10.1002/jimd.12503.
3. Storgaard, J.H.; Løkken, N.; Madsen, K.L.; Voermans, N.C.; Laforêt, P.; Nadaj-Pakleza, A.; Tard, C.; van Hall, G.; Vissing, J.; Ørngreen, M.C. No Effect of Resveratrol on Fatty Acid Oxidation or Exercise Capacity in Patients with Fatty Acid Oxidation Disorders: A Randomized Clinical Cross-over Trial. *J. Inherit. Metab. Dis.* **2022**, *45*, 517–528, doi:10.1002/jimd.12479.

4. Norris, M.K.; Scott, A.I.; Sullivan, S.; Chang, I.J.; Lam, C.; Sun, A.; Hahn, S.; Thies, J.M.; Gunnarson, M.; McKean, K.N.; et al. Tutorial: Triheptanoin and Nutrition Management for Treatment of Long-Chain Fatty Acid Oxidation Disorders. *J. Parenter. Enter. Nutr.* **2021**, *45*, 230–238, doi:10.1002/jpen.2034.
5. Rücklová, K.; Hrubá, E.; Pavlíková, M.; Hanák, P.; Farolfi, M.; Chrastina, P.; Vlášková, H.; Kousal, B.; Smolka, V.; Foltenová, H.; et al. Impact of Newborn Screening and Early Dietary Management on Clinical Outcome of Patients with Long Chain 3-Hydroxyacyl-CoA Dehydrogenase Deficiency and Medium Chain Acyl-CoA Dehydrogenase Deficiency—A Retrospective Nationwide Study. *Nutrients* **2021**, *13*, 2925, doi:10.3390/nu13092925.
6. Guffon, N.; Mochel, F.; Schiff, M.; De Lonlay, P.; Douillard, C.; Vianey-Saban, C. Clinical Outcomes in a Series of 18 Patients with Long Chain Fatty Acids Oxidation Disorders Treated with Triheptanoin for a Median Duration of 22 Months. *Mol. Genet. Metab.* **2021**, *132*, 227–233, doi:10.1016/j.ymgme.2021.02.003.
7. Stenlid, R.; Olsson, D.; Cen, J.; Manell, H.; Haglind, C.; Chowdhury, A.I.; Bergsten, P.; Nordenström, A.; Halldin, M. Altered Mitochondrial Metabolism in Peripheral Blood Cells from Patients with Inborn Errors of B-oxidation. *Clin. Transl. Sci.* **2022**, *15*, 182–194, doi:10.1111/cts.13133.
8. Zöggeler, T.; Stock, K.; Jörg-Streller, M.; Spenger, J.; Konstantopoulou, V.; Hufgard-Leitner, M.; Scholl-Bürgi, S.; Karall, D. Long-Term Experience with Triheptanoin in 12 Austrian Patients with Long-Chain Fatty Acid Oxidation Disorders. *Orphanet J. Rare Dis.* **2021**, *16*, 28, doi:10.1186/s13023-020-01635-x.
9. Vockley, J.; Burton, B.; Berry, G.T.; Longo, N.; Phillips, J.; Sanchez-Valle, A.; Tanpaiboon, P.; Grunewald, S.; Murphy, E.; Humphrey, R.; et al. UX007 for the Treatment of Long Chain-Fatty Acid Oxidation Disorders: Safety and Efficacy in Children and Adults Following 24 Weeks of Treatment. *Mol. Genet. Metab.* **2017**, *120*, 370–377, doi:10.1016/j.ymgme.2017.02.005.
10. Vockley, J.; Burton, B.; Berry, G.T.; Longo, N.; Phillips, J.; Sanchez-Valle, A.; Tanpaiboon, P.; Grunewald, S.; Murphy, E.; Bowden, A.; et al. Results from a 78-week, Single-arm, Open-label Phase 2 Study to Evaluate UX007 in Pediatric and Adult Patients with Severe Long-chain Fatty Acid Oxidation Disorders (LC-FAOD). *J. Inherit. Metab. Dis.* **2019**, *42*, 169–177, doi:10.1002/jimd.12038.
11. Lee, S.K.; Gupta, M.; Shi, J.; McKeever, K. The Pharmacokinetics of Triheptanoin and Its Metabolites in Healthy Subjects and Patients With Long-Chain Fatty Acid Oxidation Disorders. *Clin. Pharmacol. Drug Dev.* **2021**, *10*, 1325–1334, doi:10.1002/cpdd.944.
12. Anderson, D.R.; Viau, K.; Botto, L.D.; Pasquali, M.; Longo, N. Clinical and Biochemical Outcomes of Patients with Medium-Chain Acyl-CoA Dehydrogenase Deficiency. *Mol. Genet. Metab.* **2020**, *129*, 13–19, doi:10.1016/j.ymgme.2019.11.006.
13. Gillingham, M.B.; Elizondo, G.; Behrend, A.; Matern, D.; Schoeller, D.A.; Harding, C.O.; Purnell, J.Q. Higher Dietary Protein Intake Preserves Lean Body Mass, Lowers Liver Lipid Deposition, and Maintains Metabolic Control in Participants with Long-chain Fatty Acid Oxidation Disorders. *J. Inherit. Metab. Dis.* **2019**, *42*, 857–869, doi:10.1002/jimd.12155.
14. Rovelli, V.; Manzoni, F.; Viau, K.; Pasquali, M.; Longo, N. Clinical and Biochemical Outcome of Patients with Very Long-Chain Acyl-CoA Dehydrogenase Deficiency. *Mol. Genet. Metab.* **2019**, *127*, 64–73, doi:10.1016/j.ymgme.2019.04.001.
15. Shiraishi, H.; Yamada, K.; Oki, E.; Ishige, M.; Fukao, T.; Hamada, Y.; Sakai, N.; Ochi, F.; Watanabe, A.; Kawakami, S.; et al. Open-Label Clinical Trial of Bezafibrate Treatment in Patients with Fatty Acid Oxidation Disorders in Japan; 2nd Report QOL Survey. *Mol. Genet. Metab. Rep.* **2019**, *20*, 100496, doi:10.1016/j.ymgmr.2019.100496.
16. Yamada, K.; Shiraishi, H.; Oki, E.; Ishige, M.; Fukao, T.; Hamada, Y.; Sakai, N.; Ochi, F.; Watanabe, A.; Kawakami, S.; et al. Open-Label Clinical Trial of Bezafibrate Treatment in Patients with Fatty Acid Oxidation Disorders in Japan. *Mol. Genet. Metab. Rep.* **2018**, *15*, 55–63, doi:10.1016/j.ymgmr.2018.02.003.
17. Bleeker, J.C.; Kok, I.L.; Ferdinandusse, S.; de Vries, M.; Derks, T.G.J.; Mulder, M.F.; Williams, M.; Gozalbo, E.R.; Bosch, A.M.; van den Hurk, D.T.; et al. Proposal for an Individualized Dietary Strategy in Patients with Very Long-chain Acyl-CoA Dehydrogenase Deficiency. *J. Inherit. Metab. Dis.* **2019**, *42*, 159–168, doi:10.1002/jimd.12037.

18. MacDonald, A.; Webster, R.; Whitlock, M.; Gerrard, A.; Daly, A.; Preece, M.A.; Evans, S.; Ashmore, C.; Chakrapani, A.; Vijay, S.; et al. The Safety of Lipistart, a Medium-Chain Triglyceride Based Formula, in the Dietary Treatment of Long-Chain Fatty Acid Disorders: A Phase I Study. *J. Pediatr. Endocrinol. Metab.* **2018**, *31*, 297–304, doi:10.1515/jpem-2017-0426.
19. Gillingham, M.B.; Heitner, S.B.; Martin, J.; Rose, S.; Goldstein, A.; El-Gharbawy, A.H.; Deward, S.; Lasarev, M.R.; Pollaro, J.; DeLany, J.P.; et al. Triheptanoin versus Trioctanoin for Long-Chain Fatty Acid Oxidation Disorders: A Double Blinded, Randomized Controlled Trial. *J. Inherit. Metab. Dis.* **2017**, *40*, 831–843, doi:10.1007/s10545-017-0085-8.
20. De Biase, I.; Viau, K.S.; Liu, A.; Yuzyuk, T.; Botto, L.D.; Pasquali, M.; Longo, N. Diagnosis, Treatment, and Clinical Outcome of Patients with Mitochondrial Trifunctional Protein/Long-Chain 3-Hydroxy Acyl-CoA Dehydrogenase Deficiency. *JIMD Rep. Vol. 31* **2016**, *31*, 63–71, doi:10.1007/8904_2016_558.
21. Evans, M.; Andresen, B.S.; Nation, J.; Boneh, A. VLCAD Deficiency: Follow-up and Outcome of Patients Diagnosed through Newborn Screening in Victoria. *Mol. Genet. Metab.* **2016**, *118*, 282–287, doi:10.1016/j.ymgme.2016.05.012.
22. Haglind, C.B.; Stenlid, M.H.; Ask, S.; Alm, J.; Nemeth, A.; Döbeln, Uv.; Nordenström, A. Growth in Long-Chain 3-Hydroxy-acyl-CoA Dehydrogenase Deficiency. *JIMD Rep. - Case Res. Rep. 20125* **2012**, *8*, 81–90, doi:10.1007/8904_2012_164.
23. Orngreen, M.C.; Madsen, K.L.; Preisler, N.; Andersen, G.; Vissing, J.; Laforet, P. Bezafibrate in Skeletal Muscle Fatty Acid Oxidation Disorders: A Randomized Clinical Trial. *Neurology* **2014**, *82*, 607–613, doi:10.1212/WNL.0000000000000118.
24. Behrend, A.M.; Harding, C.O.; Shoemaker, J.D.; Matern, D.; Sahn, D.J.; Elliot, D.L.; Gillingham, M.B. Substrate Oxidation and Cardiac Performance during Exercise in Disorders of Long Chain Fatty Acid Oxidation. *Mol. Genet. Metab.* **2012**, *105*, 110–115, doi:10.1016/j.ymgme.2011.09.030.
25. Gillingham, M.B.; Purnell, J.Q.; Jordan, J.; Stadler, D.; Haqq, A.M.; Harding, C.O. Effects of Higher Dietary Protein Intake on Energy Balance and Metabolic Control in Children with Long-Chain 3-Hydroxy Acyl-CoA Dehydrogenase (LCHAD) or Trifunctional Protein (TFP) Deficiency. *Mol. Genet. Metab.* **2007**, *90*, 64–69, doi:10.1016/j.ymgme.2006.08.002.
26. Iafolla, A.K.; Thompson, R.J.; Roe, C.R. Medium-Chain Acyl-Coenzyme A Dehydrogenase Deficiency: Clinical Course in 120 Affected Children. *J. Pediatr.* **1994**, *124*, 409–415, doi:10.1016/s0022-3476(94)70363-9.
27. Haglind, C.B.; Nordenström, A.; Ask, S.; von Döbeln, U.; Gustafsson, J.; Stenlid, M.H. Increased and Early Lipolysis in Children with Long-Chain 3-Hydroxyacyl-CoA Dehydrogenase (LCHAD) Deficiency during Fast. *J. Inherit. Metab. Dis.* **2015**, *38*, 315–322, doi:10.1007/s10545-014-9750-3.
28. Jakobs, C.; Kneer, J.; Martin, D.; Boulloche, J.; Brivet, M.; Poll-The, B.T.; Saudubray, J.M. In Vivo Stable Isotope Studies in Three Patients Affected with Mitochondrial Fatty Acid Oxidation Disorders: Limited Diagnostic Use of 1-13C Fatty Acid Breath Test Using Bolus Technique. *Eur. J. Pediatr.* **1997**, *156*, S78–S82, doi:10.1007/PL00014278.
29. Fletcher, J.M.; Pitt, J.J. Fasting Medium Chain Acyl-Coenzyme a Dehydrogenase-Deficient Children Can Make Ketones. *Metabolism* **2001**, *50*, 161–165, doi:10.1053/meta.2001.20177.
30. Gillingham, M.B.; Harding, C.O.; Schoeller, D.A.; Matern, D.; Purnell, J.Q. Altered Body Composition and Energy Expenditure but Normal Glucose Tolerance among Humans with a Long-Chain Fatty Acid Oxidation Disorder. *Am. J. Physiol.-Endocrinol. Metab.* **2013**, *305*, E1299–E1308, doi:10.1152/ajpendo.00225.2013.
31. Huidekoper, H.H.; Ackermans, M.T.; Koopman, R.; van Loon, L.J.C.; Sauerwein, H.P.; Wijburg, F.A. Normal Rates of Whole-Body Fat Oxidation and Gluconeogenesis after Overnight Fasting and Moderate-Intensity Exercise in Patients with Medium-Chain Acyl-CoA Dehydrogenase Deficiency. *J. Inherit. Metab. Dis.* **2013**, *36*, 831–840, doi:10.1007/s10545-012-9532-8.
32. Diekman, E.F.; Visser, G.; Schmitz, J.P.J.; Nievelstein, R.A.J.; de Sain-van der Velden, M.; Wardrop, M.; Van der Pol, W.L.; Houten, S.M.; van Riel, N.A.W.; Takken, T.; et al. Altered Energetics of Exercise Explain Risk of Rhabdomyolysis in Very Long-Chain Acyl-CoA Dehydrogenase Deficiency. *PLOS ONE* **2016**, *11*, e0147818, doi:10.1371/journal.pone.0147818.

33. Akamizu, T.; Sakura, N.; Shigematsu, Y.; Tajima, G.; Ohtake, A.; Hosoda, H.; Iwakura, H.; Ariyasu, H.; Kangawa, K. Analysis of Plasma Ghrelin in Patients with Medium-Chain Acyl-CoA Dehydrogenase Deficiency and Glutaric Aciduria Type II. *Eur. J. Endocrinol.* **2012**, *166*, 235–240, doi:10.1530/EJE-11-0785.
34. McCoin, C.S.; Piccolo, B.D.; Knotts, T.A.; Matern, D.; Vockley, J.; Gillingham, M.B.; Adams, S.H. Unique Plasma Metabolomic Signatures of Individuals with Inherited Disorders of Long-Chain Fatty Acid Oxidation. *J. Inherit. Metab. Dis.* **2016**, *39*, 399–408, doi:10.1007/s10545-016-9915-3.
35. Madsen, K.L.; Stemmerik, M.G.; Buch, A.E.; Poulsen, N.S.; Lund, A.M.; Vissing, J. Impaired Fat Oxidation During Exercise in Long-Chain Acyl-CoA Dehydrogenase Deficiency Patients and Effect of IV-Glucose. *J. Clin. Endocrinol. Metab.* **2019**, *104*, 3610–3613, doi:10.1210/jc.2019-00453.
36. Madsen, K.L.; Preisler, N.; Buch, A.E.; Stemmerik, M.G.; Laforêt, P.; Vissing, J. Impaired Fat Oxidation during Exercise in Multiple Acyl-CoA Dehydrogenase Deficiency. *JIMD Rep.* **2019**, *46*, 79–84, doi:10.1002/jmd2.12024.
37. McCoin, C.S.; Gillingham, M.B.; Knotts, T.A.; Vockley, J.; Ono-Moore, K.D.; Blackburn, M.L.; Norman, J.E.; Adams, S.H. Blood Cytokine Patterns Suggest a Modest Inflammation Phenotype in Subjects with Long-chain Fatty Acid Oxidation Disorders. *Physiol. Rep.* **2019**, *7*, e14037, doi:10.14814/phy2.14037.
